# The Med-NKQ: A Reliable Mediterranean Diet Nutrition Knowledge Questionnaire for Cardiovascular Disease

**DOI:** 10.3390/nu13092949

**Published:** 2021-08-25

**Authors:** Carissa Moroney, Fiona O’Leary, Victoria M. Flood

**Affiliations:** 1 Sydney School of Health Sciences, Faculty of Medicine and Health, The University of Sydney, Camperdown, NSW 2006, Australia; cmor3553@uni.sydney.edu.au; 2 Sydney School of Nursing, Faculty of Medicine and Health, The University of Sydney, Camperdown, NSW 2006, Australia; fiona.oleary@sydney.edu.au; 3 Western Sydney Local Health District, Research and Education Network, Westmead Hospital, Westmead, NSW 2145, Australia

**Keywords:** mediterranean diet, nutrition knowledge, cardiovascular disease, repeatability, delphi, survey

## Abstract

The Mediterranean diet (MD) has significant benefits for cardiovascular disease (CVD), yet clinicians lack reliable tools to measure patient knowledge. This study aimed to develop a short tool to test knowledge of MD related to cardiac health. Themes included foods to reduce CVD risk factors, quantification of servings, and common MD dietary patterns; a maximum score of 42 was determined for correct responses. Content validity was assessed through expert consensus in a Delphi survey. A 70% level of agreement was set for each domain tested. Repeatability was assessed via a test-retest protocol in a sample with self-reported CVD, advertised through social media and administered online. Ten and six of twenty-five invited experts responded to round one and two of the Delphi survey respectively. All items achieved greater than 70% consensus. Twenty people completed the repeatability study. A paired *t*-test found no significant difference in mean scores between the two test periods (Test one, 28 (standard deviation (SD) 5.4). Test two 29.5, (SD 5.5), *p* = 0.174) and a Bland-Altman Plot indicated no bias between the two surveys. The Med-NKQ demonstrated good content validity and reliability in people with CVD, and is short and easy to administer, making it practical in clinical and research settings.

## 1. Introduction

Cardiovascular disease (CVD) is the number one cause of death worldwide [1]. Approximately 30% of all global deaths are attributed to the disease, many of which are considered preventable through lifestyle modification [1,2,3]. The Mediterranean diet has demonstrated benefits for the prevention of both primary and secondary cardiac events [4,5,6]. Although definitions vary, a traditional definition of the Mediterranean diet is regarded as one that is mostly plant based, high in vegetables, especially leafy greens, fruit, whole grains, and legumes; moderate in dairy; and low in meat products. Fish consumption is based on proximity to the sea and the main culinary oil is extra virgin olive oil, used liberally, and moderate red wine consumption, with meals, if religious beliefs allow [7]. It has been suggested that whilst many of these individual components of the diet have demonstrated benefits for cardiac health, the combined effects of the dietary pattern as a whole diet approach provide additional protective benefits [8].

Adopting a Mediterranean diet pattern for the improvement of cardiac health requires an individual to undergo a behaviour change process, which is multifactorial, including the development of both knowledge and skills, which are more likely to be maintained in a supportive environment [9,10]. This is often achieved through group education programs such as those delivered within cardiac rehabilitation. Short item dietary screeners including the 14-point Mediterranean Diet Adherence Screener (MEDAS) used within the PREDIMED trial and more recently the 17-point energy-restricted Mediterranean Diet Adherence Screener (er-MEDAS) are validated measures of dietary adherence, which, due to their short form, are ideal for use in a clinical setting [11,12]. Unfortunately, nutrition knowledge is rarely evaluated, and this may in part be due to the lack of appropriate tools for use in clinical practice [13]. This represents a large gap in the evaluation of health education programs because nutrition knowledge is a required precursor to behaviour change that predicts adherence [14,15,16].

Despite significant interest in the Mediterranean diet for CVD prevention, there are few tools to assess knowledge of the Mediterranean diet and no tools that assess Mediterranean diet knowledge specific to cardiac health [16,17,18]. Previously validated tools such as the General Nutrition Knowledge Questionnaire (GNKQ) do not test aspects of the Mediterranean diet and due to their length and generic nature are impractical for routine use in clinical practice [19].

Therefore, the aim of this study was to develop and assess the reliability of a short Mediterranean diet nutrition knowledge tool, specific to cardiac health and suitable for routine use in a clinical setting.

## 2. Materials and Methods

### 2.1. Development of Mediterranean Diet Nutrition Knowledge Questionnaire (Med-NKQ)

A 20-item tool was developed by the research team with clinical and academic expertise in cardiac nutrition, the Mediterranean diet, and survey design. Instrument construction was informed by previously published systematic reviews, meta-analyses, randomized controlled trials, prospective cohort studies, position papers, and best practice guidelines from peak cardiac health and nutrition bodies, national dietary guidelines (Table S1), and clinical experience leading CVD group education programs, including knowledge of commonly observed dietary misconceptions [3,5,20,21,22,23,24,25,26,27,28,29,30,31,32,33,34,35,36,37,38,39,40,41,42,43,44,45,46,47,48,49,50,51,52,53,54,55].

Questions were in multiple-choice format and 11 (55%) included images to enhance participant engagement and support comprehension of the written text. The tool focused on three major themes: (1) Dietary patterns, foods, and nutrients for reducing cardiac risk factors such as hypertension and hyperlipidaemia (four questions); (2) testing of ability to quantify serving sizes, read labels and identify key cardioprotective nutrients (seven questions); and (3) Core Mediterranean diet foods, dietary patterns, and meal selection (nine questions). Items varied in complexity and scoring was weighted accordingly. The scoring system used was based on similarly designed validated nutrition knowledge tools and is available in the Supplementary Material Table S2 [56,57]. For questions that only had one correct answer, one point was awarded for a question with low difficulty, two points were awarded for a question with moderate difficulty, and three points were awarded for complex or difficult questions. For questions with multiple correct answers, one point was awarded per correct response and one point was deducted per incorrect response. Negative scores were adjusted to a zero for each question to avoid awarding negative points. In total, the 20-item tool has a possible score range from 0 to 42, with a higher score indicating greater knowledge. The tool was designed to take approximately 15 min to complete.

### 2.2. Phase 1: Content Validity

A Delphi survey was used to gain expert feedback on the 20-item draft tool. The Delphi method uses multiple rounds of structured evaluation to gain feedback from experts, including ranking the relevance or importance of items, ultimately leading to a consensus or agreement on the inclusion, exclusion, or modification of a question item [58,59]. Feedback is collated, items amended, and revised items sent back to the experts for a second round and repeated until consensus is achieved. To minimise bias, experts remain anonymous to each other. In this study, experts were asked to provide feedback on three domains. Domain one asked if they agreed the answer provided for each item was correct (possible responses: agree, disagree, unsure). Domain two asked whether they felt the item was relevant for assessing knowledge of the Mediterranean diet and/or cardiac nutrition (possible responses: highly relevant, quite relevant, somewhat relevant, not relevant). The third domain asked if they would keep, remove or modify the question (possible responses: retain, delete, modify). Experts were also given the opportunity to provide free text comments on items, including suggested modifications.

A level of agreement of 70% was set for each of the three domains described above [60]. If any of the 20 knowledge questions did not achieve 70% or greater agreement from respondents in all three domains, it was removed or modified prior to proceeding to round two of the Delphi.

Prospective participants with significant clinical experience and/or research expertise in the field of cardiac nutrition and/or Mediterranean diet were identified by the research team. This included senior practicing dietitians working within clinical cardiac specialties, including cardiac rehabilitation programs and nutrition researchers publishing within the Mediterranean diet and/or cardiac health fields. A target sample of 25 experts (clinical: *n* = 10; research: *n* = 15) were contacted by email to invite them to participate in the Delphi survey. Practicing clinicians and academic researchers were invited to facilitate feedback from both clinical and academic perspectives. Consent to participate in the study was implied via return of the completed survey. Study approval was granted by the University of Sydney Human Research Ethics Committee, approval number 2017/617.

### 2.3. Phase 2: Testing Repeatability of the Med-NKQ

Following the Delphi survey, the finalised tool was tested for repeatability using a test-retest study design and was administered via REDCap, hosted at The University of Sydney (version 10.0.1) as an online survey, due to timing of testing coinciding with the COVID-19 global pandemic, resulting in face-to-face recruitment being unsuitable.

A short questionnaire to obtain demographic information preceded the 20-item knowledge tool. The study was advertised using social media platforms including Facebook and Twitter, as well as community newsletters. Consent was obtained from participants though the online survey and the study was approved by the University of Sydney Human Research Ethics Committee, approval number 2020/338.

Participants were included in the study if they could read and write English, were 18 years or older, and self-reported a pre-existing cardiac disease or previous cardiac surgery. A total of 32 participants were recruited and 20 completed the study. During the completion of the online tool, participants provided their email address and a 2-week waiting period occurred prior to an automated email being sent to provide a link for completion of the second round of the tool. Data was analysed using SPSS software version 27.

## 3. Results

### 3.1. Phase 1: Content Validity

**Figure 1:**
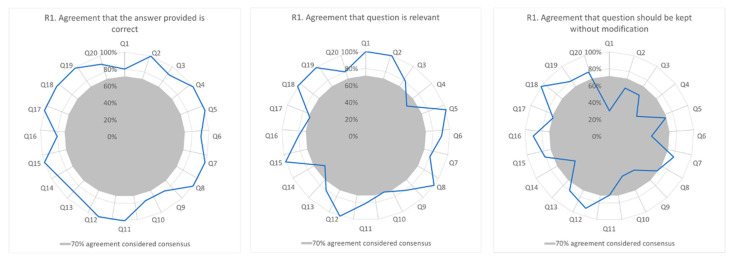
Delphi Round 1 participant responses (*n* = 10), in relation to agreement of answer, relevancy, and whether modification is required. Q= question number.

Of the 25 experts contacted, 10 responded (clinical: *n* = 3; research: *n* = 7), giving a response rate of 40% for Delphi round one. Results pertaining to agreement with item answer, relevance to the Mediterranean diet, and need for modification are summarised in Figure 1.

All 20 items achieved a group consensus ≥ 70% (range 80 to 100%) that the answers indicated on the instrument were correct. Minor disagreements were noted for six items. Question 1 about cholesterol-lowering foods had disagreement from two participants and question 6, regarding sources of healthy fats, had disagreement from one participant. In a further four questions (questions 3, 9, 10, and 14), one person indicated they were “unsure” that the correct answer provided by the researchers was correct.

In relation to relevance to the Mediterranean diet, 18 out of the 20 items developed were considered highly relevant or quite relevant by more than 70% of respondents. The two questions that did not reach consensus were question 4, which asked true/false statements, and question 14, which asked about cooking techniques relevant to the Mediterranean diet.

In 60% of the items there was consensus that the question should be retained without modification. Eight questions did not reach 70% consensus and therefore were modified. Suggested modifications ranged from small changes in sentence wording to more significant recommendations such as choosing different foods to represent sources of saturated and unsaturated fat in a question relating to sources of fat for cardiac health.

Overall changes were made to 13 (65%) questions. Changes made are described in Supplementary Material Table S3. Minor changes to wording and terminology were made to eight questions, whilst a further five underwent greater modification, with one question completely modified on the advice that traditional beverages with the exception of alcohol had not been considered.

After modification, version two of the Delphi was created. This was returned to the 10 participants who responded in round one along with the anonymous comments from round one experts to give context to the changes made. The respondents were then asked to provide feedback on version two of the tool.

**Figure 2:**
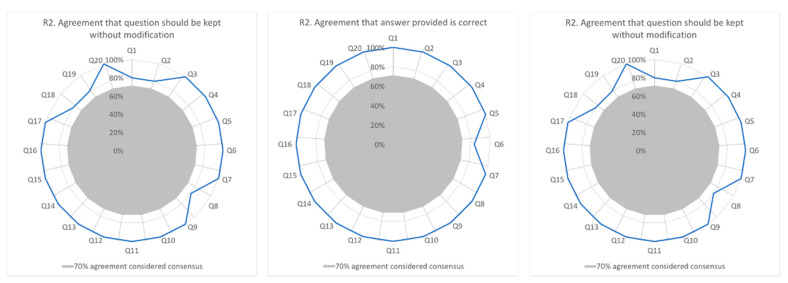
Delphi Round 2 participant responses (*n* = 6), in relation to agreement of answer, relevancy, and whether modification is required. Q= question number.

Of the 10 participants contacted in round two, six (60%) (clinical: *n* = 2; research *n* = 4) responded. Results pertaining to agreement with item answer, relevance to the Mediterranean diet, and need for modification are summarised in Figure 2. 

All items, with the exception of one (question 6, 83% agreement) relating to sources of healthy fats, received 100% agreement for correct response. One participant raised an issue regarding unclear evidence surrounding coconut oil [44]. With respect to relevance, all items reached > 70% agreement although two questions (3 and 18) relating to servings of vegetables and selecting meals reflective of the Mediterranean diet respectively had one participant who indicated the items were only somewhat relevant. With respect to keeping or modifying items, a consensus of >70% was achieved for all items in the tool with five items having one participant each suggesting a recommendation for modification. These modifications ranged from a change in wording to a comment that some items might be easy to ‘guess’ based on the wording of the question. One item (question 6; agreement that the answer provided is correct) achieved less consensus in round two than round one due to the smaller sample size for round two, which altered the expressed percentages (round one: 9 out of 10 experts agreed (90%); compared to round two, 5 out of 6 experts agreed (83%)). Question 18, agreement that the answer should be kept without modification, observed 100% agreement in round one and 83% agreement in round two, as one participant changed their response. On the basis of the overall high level of agreement for the tool in round two, no further rounds were undertaken, and the tool was finalised. The finalised tool was named Med-NKQ and is available in the Supplementary Material Table S4.

### 3.2. Phase Two: Repeatability

Thirty-two participants completed the Med-NKQ on the first occasion (test one) and 20 people proceeded to complete the tool a second time (test two). Descriptive statistics are shown in Table 1. Respondents were predominantly male (60%), aged 50 or older (90%), had high school level education or higher (100%), and were Australian born (80%). Half of the group self-reported a history of coronary heart disease (blocked or narrowed vessels of the heart) with 70% of respondents undergoing some form of cardiac surgery for their condition including cardiac stent (45%), coronary artery bypass surgery (15%), or cardiac valve replacement (10%).

A paired sample *t*-test showed no significant difference in the mean total scores of test one (28, standard deviation (SD) 5.4) compared to test two (29.5, SD 5.5) (*t* = 1.41, *p* = 0.174). Pearson’s correlation was significant between the two test scores (*r* = 0.59, *p* = 0.006). A power analysis confirms 20 pairs are adequately powered to detect the mean of the differences of 10%, for a mean correct score of 66% correct (i.e., 28/42) (80% power, two sided *p*-value < 0.05). Ten percent mean of the differences was considered clinically relevant.

**Figure 3:**
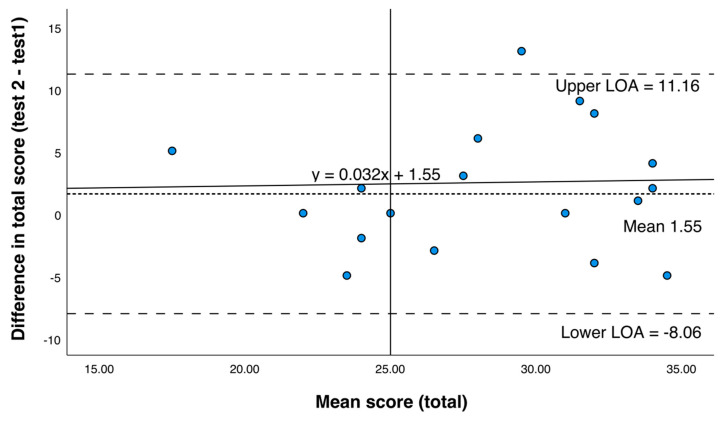
Bland Altman Plot assessing the reliability of Med-NKQ-20 during test re-test. The plot shows the mean difference (^…..…..^), 95% limits of agreement (LOA, - - - - - - - ), and fitted regression line (^_______^), for overall test score (test 2-test 1), β = 0.032, *t* = 0.136 *p* = 0.893, 95% CI (−0.469, 0.535), equation of line y = 0.032x + 1.55.

The Bland Altman plot shows most measures falling within the 95% limit of agreement with the fitted regression line (y = 0.032x + 1.55), indicating no bias between the two attempts (Figure 3).

Subgroup analysis of questionnaire themes found no significant difference in the mean scores of test one and test two for two of the three subgroups (nutrient content of foods; quantification and label reading; and core MD foods, patterns, and meal selection) (Table 2). However, a significant difference was detected between the mean of test one and test two for the subgroup analysis of questions relating to risk factors (questions 1, 2, 4, and 5): mean score test one, 7.5 (SD 1.9); mean score test two, 8.3 (SD 1.3) *p* = 0.018.

There were three questions where less than 50% of respondents scored full points (questions 1, 8 and 14). Question 1 asked respondents to select true statements regarding dietary strategies for reducing cholesterol. Eleven (55%) and 13 (65%) respondents respectively in test one and test two incorrectly selected reducing salt consumption as a strategy to reduce cholesterol. Question 8 also related to salt consumption and was a pictorial question asking respondents to correctly identify foods high in salt. Fifteen (75%) and 11 (55%) participants respectively in test one and test two were unable to correctly identify smoked salmon as high in salt. Question 14 was related to cooking methods relevant to the Mediterranean diet and although 13 (65%) respondents in both test one and two were correctly able to identify moist cooking methods such as stewing as the most typical cooking method, 14 (70%) respondents in test one and 18 (90%) respondents in test two also incorrectly selected additional cooking methods, resulting in loss of points.

There were eight questions where 75% or more of respondents scored full points (questions 5, 7, 10, 12, 13, 18, 19, and 20). All of these questions were pictorial, with question 19 and 20 being label-reading questions.

## 4. Discussion

This study demonstrated that the Med-NKQ is a reliable tool for assessing nutrition knowledge relevant to the Mediterranean diet and cardiac health. The tool demonstrated good content validity through expert consensus and good repeatability in a test re-test study. The 20-item tool has a possible score range from 0 to 42, with a higher score indicating greater knowledge. The tool, designed to be used in group education programs such as cardiac rehabilitation, takes approximately 15 min to complete and covers three themes of nutrition knowledge related to CVD risk factors, nutrient content of foods, and dietary patterns consistent with the Mediterranean diet. Three previous studies reported the use of a Mediterranean diet nutrition knowledge tool [16,17,18]. However, to the authors knowledge, this is the first Mediterranean diet nutrition knowledge tool designed with a focus on cardiac health. Tools designed by Tsartsali and Sahingoz were designed for use in an adolescent population and are not suitable to evaluate nutrition knowledge in an adult cohort of patients with CVD. The tool designed by Bottcher et al., whilst brief at 15 questions, making it suitable for the clinical environment, and designed for an adult population, includes complex questions surrounding caloric content of foods whilst placing less emphasis on whole foods, traditional approaches, and diet patterns used within the Mediterranean Diet. This implies that an individual must have sound understanding of energy density rather than an understanding of types of foods and cooking methods to have useful knowledge of the Mediterranean diet. This is in contrast to the findings of Grosso et al. that the benefit of the Mediterranean Diet lies in its whole diet approach rather than a focus on single nutrients [8].

Dietary behaviour change is a complex yet essential intervention to reduce the health and economic burden of CVD [1,61,62]. Whilst improving nutrition knowledge is only one component of the behaviour change process, it has been said that ‘one must know before one can do [63]. With growing evidence to support the imbedding of the Mediterranean diet into cardiac group education programs, clinicians and researchers alike require appropriate tools to evaluate the effectiveness of interventions [21,22]. The Med-NKQ complements dietary adherence screeners, recently endorsed by the American Heart Association, through diversifying the tools available to provide a more comprehensive assessment of dietary intervention educational effects [11,12,64].

We achieved a high level of consensus after only two rounds of the Delphi survey, indicating good agreement amongst the experts. However, there were differences noted amongst experts that were of interest to the researchers. Cooking technique was seen to have low relevance to some experts whilst being rated as highly relevant by others. This was further reflected in the repeatability study, with the cooking methods question being one of the most poorly answered questions by respondents. This may be due to cooking technique, whilst having great importance to the Mediterranean diet, not being as well described in the literature. The traditional methods of low temperature, moist cooking result in the reduction of advanced glycation end products known to be harmful to cardiac health and therefore should be considered as an important topic of education, and hence assessed in the Med-NKQ [27,40,54].

Coconut oil as a source of unhealthy fat was a controversial topic for one respondent, with recent emerging but limited evidence that it may not have the same negative health effects as other saturated fats [44]. Whilst the position of international cardiac and nutrition bodies remains that coconut oil should be avoided, it was removed from the tool to minimise contention as future research emerges [65,66,67,68]. One image change and two additional wording changes were made to the Med-MKQ (shown in Table S2), which were not re-tested among the experts in the Delphi survey, and we acknowledge this as a limitation of the work.

Pictorial questions were more likely to be answered correctly by participants in the repeatability study with evidence suggesting surveys and written text designed with visual aids are completed in less time and with greater participant comprehension [69,70]. A significant difference was detected in change of score for CVD risk factors between the two test periods. A known limitation of the test re-test method is that participants may conduct their own learning between test periods, even if they are requested not to [57]. To minimise this influence, the time period between the two test periods of 2 weeks, as suggested by Trackman et al., was adopted for this study [57]. It is possible this significant finding could be related to the known limitation of this methodology. Further testing of the Med-NKQ within an intervention study may help to determine the clinical significance of this finding.

Due to the short 20-question format, this tool does not fully examine all aspects of the Mediterranean diet and its concepts. However, this tool was designed to increase uptake of evaluation tools in a time- and resource-limited clinical environment. Whilst it is not entirely comprehensive, instruments for assessing patients in the clinical setting have rapid uptake when they are short and easy to administer [71,72].

It was estimated that only 35% of nutrition education programs evaluate patient knowledge. This may be in part due to the lack of reliable tools available to clinicians and researchers [13]. It is expected that the Med-NKQ presented in this paper will lead to higher quality education programs through improvements in evaluation and hence better outcomes for patients with CVD.

Further testing of the Med-NKQ within other population groups will strengthen the utility of the tool. Participants within this study were more likely to be educated beyond secondary school level (*n* = 12, 60%) and further testing among people with a lower level of education will broaden the application of the Med-NKQ. It would be useful to assess change in Med-NKQ scores following education programs, among people with CVD, and compare to other cohorts, such as the general older population and health professional groups. This would further increase the application of the Med-NKQ in clinical practice and intervention studies.

Whilst this tool was developed for the Australian environment, using National Health and Medical Research Council (NHMRC) recommendations for serving sizes, and Food Standards Australia New Zealand (FSANZ) standards for nutrition information panels and units of measure, the Med-NKQ has the capacity to be modified to suit a wider international audience by altering units of measure and translating recommended serving sizes to relevant national recommendations.

Although the sample size of the Delphi study was small, particularly the second round, the literature suggests that smaller sample sizes may not be a limitation of the Delphi method [58,73]. The experts who dropped out at the end of round one did not account for the majority of disagreement observed, however we cannot rule out the possibility of some degree of bias as a result of a reduced number of experts who participated in the second round.

Similar to the Delphi survey, the repeatability study also had a small sample size. However, a power analysis confirms the study is adequately powered to detect a clinically significant change. Previously published repeatability studies of similar design examining the validity of various nutrition knowledge tools have reported comparable sample sizes. [56,74,75,76].

It is likely the small sample size is related to the online recruitment method used in this study and the need to recruit from people who had CVD. The COVID-19 global pandemic that occurred during the recruitment phase of this study resulted in the cessation of face-to-face group education programs, which was the intended source of recruitment. It is likely that the online recruitment method resulted in a younger than expected demographic of respondents, 55% in the 50–59 age range. Although online recruitment may have impacted sample size, the successful online administration of the questionnaire can be seen as a strength. The global pandemic has changed the way healthcare is delivered and it is anticipated that health education programs will increasingly be delivered through remote methods such as ehealth and telehealth [77,78]. This study has demonstrated that the Med-NKQ has good application in an online environment.

## 5. Conclusions

In summary, this research provides a novel Med-NKQ, informed by the evidence, and had good content validity as assessed by experts. The Med-NKQ provides repeatable assessment of knowledge on Mediterranean diet as related to cardiac health among people with CVD, in a short, easy-to-use format with visual aids. The Med-NKQ is available freely and will contribute to future clinical practice and research interventions.

## Figures and Tables

**Table 1:** Demographic information of participants who participated in both tests of the survey (*n* = 20).

Characteristics	(*n*)	%
Age Range (years)		
30–49	2	10
50–69	11	55
70+	7	35
Gender		
Male	12	60
Female	8	40
Education level		
Secondary school	8	40
Trade certificate	6	30
University degree	6	30
Country of birth		
Australia	16	80
Other	4	20
Country of residence		
Australia	20	100
Employment		
Full time	5	25
Part time	5	25
Retired	10	50
Self-reported heart condition *		
Angina	3	15
Blocked/narrowed vessels of the heart	10	50
Heart attack	7	35
Heart bypass	3	15
Heart stent	9	45
Valve replacement	2	10

* More than one option could be selected by participants.

**Table 2:** Scoring of Med-NQK based on themes.

Themes	Related Questions	Points per Theme	Test 1 Mean Score (SD)	Test 2 Mean Score (SD)	*p* *
CVD ^†^ risk factors	1, 2, 4, 5	10	7.5 (1.9)	8.3 (1.3)	0.018
Nutrient content of foods, quantification, label reading	3, 6, 7, 8, 9, 19, 20	15	10.0 (2.5)	10.3 (2.7)	0.655
Core MD ^‡^ foods, patterns and meal selection	10, 11, 12, 13, 14, 15, 16, 17, 18	17	11.1 (3.2)	11.0 (2.8)	0.745
Total			28 (5.4)	29.5 (5.5)	0.174

* Paired t-test. ^†^ Cardiovascular disease, ^‡^ Mediterranean diet.

## Data Availability

The data presented in this study are available on request from the corresponding author. The data are not publicly available due to ethical and privacy restrictions.

## Supplementary Materials

The following are available online at https://www.mdpi.com/article/10.3390/nu13092949/s1, Table S1: Evidence supporting development of Med-NKQ. Table S2: Changes made to Med-NKQ throughout Delphi Method. Table S3: Med-NKQ Final Version. Table S4: Med-NKQ Scoring Sheet
