# Supplementary material for: The Med-NKQ: A Reliable Mediterranean Diet Nutrition Knowledge Questionnaire for Cardiovascular Disease
Source: Nutrients. 2021 Aug 25;13(9):2949. doi: 10.3390/nu13092949 (PMC8471340; doi:10.3390/nu13092949)
Supplement: Supplementary file 1 [file nutrients-13-02949-s001.zip › nutrients-1312328-supplementary.pdf]

# Supplementary Materials

**Table S1.** Evidence supporting development of Med-NKQ.

| Author (Date) | Publication type | Title | Related Med-NKQ questions | Supporting evidence informing Med-NKQ |
|---|---|---|---|---|
| Ambrosetti (2020) | Position paper | Secondary prevention through comprehensive cardiovascular rehabilitation: From knowledge to implementation. 2020 update. A position paper from the Secondary Prevention and Rehabilitation Section of the European Association of Preventive Cardiology | 1,2,4-18 | Supports implementation of MD‡ for cardiac rehabilitation patients |
| Anderson (2002) | Meta-analysis | Pulses and lipaemia, short- and long-term effect: potential in the prevention of cardiovascular disease | 7,11,17,18 | Pulses reduce CVD* risk through cholesterol, blood pressure, body weight and glycaemia improvements |
| Aune (2016) | Meta-analysis | Whole grain consumption and risk of cardiovascular disease, cancer, and all cause and cause specific mortality: systematic review and dose-response meta-analysis of prospective studies | 1,4,7,10,17,20 | 210-225 g/day wholegrain intake associated with reduced risk CVD* |
| Barrett (2019) | Systematic review | Whole grain, bran and cereal fibre consumption and CVD: a systematic review | 1,4,7,10,17,20 | Wholegrains, particularly fibre component, protective against CVD* |
| Becerra-Tomas (2019) | Meta-analysis | Legume Consumption and Cardiometabolic Health | 7,11,17 | Significant inverse association between total legume consumption and CVD* and CHD† |
| Birlouez-Aragon (2010) | RCT | A diet based on high-heat-treated foods promotes risk factors for diabetes mellitus and cardiovascular diseases | 14,17 | A diet that is based on high-heat-treated foods increases markers associated with an enhanced risk of CVD* in healthy people |
| Bonaccio (2017) | Prospective cohort | Fish intake is associated with lower cardiovascular risk in a Mediterranean population: Prospective results from the Moli-sani study | 6,8 | Fish intake ≥4 serves per week as part of a MD‡ reduces CHD† incidents |
| Bower (2016) | Review | The Health Benefits of Selected Culinary Herbs and Spices Found in the Traditional Mediterranean Diet | 13,14 | Use of herbs in MD‡ cuisine associated with benefits for inflammation, hyperlipidaemia and hypertension |
| Campos (2011) | Systematic Review | Nutrition labels on pre-packaged foods: a systematic review | 19,20 | Individuals with healthier eating habits report greater use of nutrition labels |

| | | | | |
|---|---|---|---|---|
| Clifton (2017) | Systematic Review | Dietary fats and cardiovascular disease: An Evidence Check rapid review brokered by the Sax Institute for the National Heart Foundation of Australia. | 1,4,5,6 | Saturated fat associated with increased mortality. Replacing saturated fats with unsaturated fats and wholegrains reduced CVD* mortality |
| Coates (2018) | Review | Nuts and Cardiovascular Disease Prevention | 6,12 | Nut consumption associated with reduced CVD* mortality and events. Blood lipids and blood pressure improved with nut consumption |
| Collins (2017) | Systematic Review | Dietary Patterns and Cardiovascular Disease Outcomes: An Evidence Check rapid review brokered by the Sax Institute (www.saxinstitute.org.au) for the National Heart Foundation of Australia | 1,2,4,5 | MD‡ improves blood lipids and CVD* events and/or mortality. MD‡ reduces blood pressure |
| Davis (2017) | RCT | A Mediterranean diet lowers blood pressure and improves endothelial function: results from the MedLey randomized intervention trial | 2,11,12,13,15, | Consumption of MD‡ for >6months resulted in significantly improved blood pressure and endothelial function. |
| Estruch (2018) | RCT | Primary Prevention of Cardiovascular Disease with a Mediterranean Diet Supplemented with Extra-Virgin Olive Oil or Nuts | 6,7,8 | MD‡ supplemented with nuts and EVOO# reduced CVD* events |
| Fatahi (2018) | RCT | Impact of Diets Rich in Whole Grains and Fruits and Vegetables on Cardiovascular Risk Factors in Overweight and Obese Women: A Randomized Clinical Feeding Trial | 1,4,5,7 | Wholegrains improve blood pressure, lipids, waist circumference and blood sugar |
| Giacosa (2104) | Review | Mediterranean Way of Drinking and Longevity | 9,16 | Moderate red wine consumption (2 glasses per day for men, 1 for women) has cardioprotective effect. Excess consumption (>89g alcohol per day) associated with increased health risks |
| Gorton (2009) | Cross-sectional | Nutrition labels: a survey of use, understanding and preferences among ethnically diverse shoppers in New Zealand | 19,20 | 82% respondents reported use of nutrition labels to inform food choice. Respondents twice as likely to use labels if have special dietary requirements, two thirds able to use Nutrition Information Panel correctly |
| He (2013) | Meta-analysis | Effect of longer-term modest salt reduction on blood pressure | 2,8,19 | Salt intakes <3g/day have significant improvements on blood pressure |
| Ho (2016) | Meta-analysis | The effect of oat beta-glucan on LDL-cholesterol, non-HDL-cholesterol and apoB for CVD risk | 7 | Oats as a source of beta-glucan fibre significantly reduced LDL§ cholesterol |

| | | | | |
|---|---|---|---|---|
| | | reduction: a systematic review and meta-analysis of randomised-controlled trials | | |
| Hoffman (2015) | Review | Food Processing and the Mediterranean Diet | 14,17 | Loss of anti-oxidant phytochemicals can occur during cooking due to thermal degradation and from leaching of substances into the cooking medium. The formation of pro-oxidants can occur when cooking at high temperatures |
| Hooper (2012) | Meta-analysis | Reduced or modified dietary fat for preventing cardiovascular disease | 1,4,5,6 | Reduction of saturated fat intake significantly reduced CVD* risk |
| Hooper (2015) | Meta-analysis | Reduction in saturated fat intake for cardiovascular disease | 1,4,5,6 | Reduction of saturated fat intake significantly reduced CVD* risk |
| Kerr (2012) | Review | Understanding standard drinks and drinking guidelines | 9 | Drinkers have difficulty defining and pouring standard drinks, intake volume is typically underestimated. |
| Khaw (2018) | RCT | Randomised trial of coconut oil, olive oil or butter on blood lipids and other cardiovascular risk factors in healthy men and women | 4,5,6 | Butter increases LDL§ compared to coconut oil and EVOO#. Coconut oil increases HDL¶ compared to butter and olive oil. Due to fatty acid profiles, coconut oil may not have the same health effects as other saturated fats in the diet |
| Kouli (2019) | Prospective cohort | Olive oil consumption and 10-year (2002-2012) cardiovascular disease incidence: the ATTICA study | 5,11,15,17 | Significant inverse association between EVOO# use and developing CVD* in a disease-free population |
| Mead (2006) | Systematic review | Dietetic guidelines on food and nutrition in the secondary prevention of cardiovascular disease – evidence from systematic reviews of randomized controlled trials (second update, January 2006) | 1,2,4-18 | Supports implementation of MD‡ for cardiac rehabilitation patients |
| Mhurchu (2007) | Review | Nutrition labels and claims in New Zealand and Australia: a review of use and understanding | 19,20 | Approximately 30-50% of consumers able to correctly interpret a food label |
| Neter (2003) | Meta-analysis | Influence of weight reduction on blood pressure: a meta-analysis of randomized controlled trials | 2 | Weight loss (>5kg) important for prevention and treatment of hypertension |
| NHMRC (2015) | National guideline | Recommended number of serves for adults | 3 | 5 serves vegetables per day recommended. Definition of a vegetable serve in Australia |
| NHMRC (2020) | National guideline | Australian guidelines to reduce health risks from drinking alcohol | 9 | Definition of a standard drink serve in Australia |

| | | | | |
|---|---|---|---|---|
| Nin (2011) | Prospective cohort | Higher Plasma Levels of Advanced Glycation End Products Are Associated With Incident Cardiovascular Disease and All-Cause Mortality in Type 1 Diabetes | 14 | Higher levels of AGE's** associated with increased incidence of CVD* |
| Oldways preservation and exchange trust (2009) | Dietary guidelines | Mediterranean Diet Pyramid | 10,11,12,13,15, 16,17,18 | MD‡ food pyramid demonstrating recommended servings of food groups according to Mediterranean Diet principles |
| Radd (2017) | Review | Evolution of Meditrranean diets and cuisine: concepts and definitions | 10,11,12,13,15, 16,17 | Definition of MD‡ and definitions of key culinary concepts |
| Sankararaman (2018) | Review | Are We Going Nuts on Coconut Oil? | 6 | Data on long term effects of coconut oil on cardiovascular health are lacking, hence coconut oil should be avoided |
| Uribarri (2010) | Laboratory study | Advanced glycation end products in foods and a practical guide to their reduction in the diet | 14 | High temperature, low moisture cooking promotes formation of AGE's**. Low temperature, high moisture and exposure to acidified environment reduce AGE** formation |
| Viguiliouk (2017) | Systematic Review | Can pulses play a role in improving cardiometabolic health? Evidence from systematic reviews and meta-analyses | 7,11,17,18 | Consumption of pulses reduces the risk of CVD* |
| Woodruffe (2015) | Review | Australian Cardiovascular Health and Rehabilitation Association (ACRA) core components of cardiovascular disease secondary prevention and cardiac rehabilitation 2014 | 1,2,3,5,6,8,9 | Reduce intake of saturated fats, salt and sugar. Increase intake of fruits and vegetables. Weight reduction for blood pressure management |

*Cardiovascular Disease , †Coronary Heart Disease , ‡Mediterranean Diet, §Low Density Lipoprotein, ¶High Density Lipoprotein, #Extra Virgin Olive Oil, **Advanced Glycation End Products.

**Table S2.** Med-NKQ Scoring Sheet.

| Question Number | Correct Answer | Scoring | Score |
|---|---|---|---|
| 1 | a) Eat more fruits and vegetables<br>b) Eat more wholegrain foods<br>c) Eat less fatty and processed meats | 1 point per correct response. -1 point for any incorrect response. 0 points if final score is negative | /3 |
| 2 | a) If using packaged foods, choose products with less salt<br>b) If you are overweight, try to use some weight<br>d) Eat more dark leafy greens | 1 point per correct response. -1 point for any incorrect response. 0 points if final score is negative | /3 |
| 3 | c) 5 serves | 1 point for single correct response | /1 |
| 4 | c) Dietary Fibre is important for lowering your cholesterol | 2 points for selecting the single correct response. -1 point for any incorrect response. 0 points if final score is negative | /2 |

| | | | |
|---|---|---|---|
| 5 | Butter<br>Fried Bacon | 1 point per correct response. -1 point for any incorrect response. 0 points if final score is negative | /2 |
| 6 | Unsalted Nuts<br>Salmon Fillet | 1 point per correct response. -1 point for any incorrect response. 0 points if final score is negative | /2 |
| 7 | Rolled Oats<br>Legumes | 1 point per correct response. -1 point for any incorrect response. 0 points if final score is negative | /2 |
| 8 | Smoked Salmon<br>Deli Ham | 1 point per correct response. -1 point for any incorrect response. 0 points if final score is negative | /2 |
| 9 | 375ml full strength beer | 3 points for single correct response | /3 |
| 10 | Pasta with tomato sauce | 1 point for single correct response | /1 |
| 11 | Legumes<br>Extra virgin olive oil | 1 point per correct response. -1 point for any incorrect response. 0 points if final score is negative | /2 |
| 12 | Leafy Greens<br>Unsalted Nuts | 1 point per correct response. -1 point for any incorrect response selected. 0 points if final score is negative | /2 |
| 13 | Oregano<br>Lemon Juice<br>Garlic | 1 point per correct response. -1 point for any incorrect response. 0 points if final score is negative | /3 |
| 14 | a) Use of moist cooking methods such as stewing | 1 point for correct response, 0 if any incorrect response | /1 |
| 15 | a) Use of Extra Virgin Olive Oil as main oil.<br>d) Mostly plant based diet. | 1 point per correct response. -1 point for any incorrect response. 0 points if final score is negative | /2 |
| 16 | a) Water | 1 point for single correct response | /1 |
| 17 | Vegetable and lentil soup with wholegrain bread and olive oil | 2 points for single correct response | /2 |
| 18 | Beans cooked in tomato and onion on toast | 1 point for single correct response | /1 |
| 19 | Canned Tuna 1 | 2 points for single correct response | /2 |
| 20 | Bread 1 | 3 points for single correct response | /3 |
| Total | | | /42 |

**Table S3.** Changes made to Med-NKQ throughout Delphi method.

| | **Description of question** | | | Changes made | |
|---|---|---|---|---|---|
| **Question Number** | **Version 1** | **Version 2** | Version 3 | Changes made Version 1 to Version 2 | Changes made Version 2 to Version 3 |
| 1 | Statements about cholesterol lowering diet patterns | Statements about cholesterol lowering diet patterns | Statements about cholesterol lowering diet patterns | Terminology changed from nutrient based to food based (e.g. from 'saturated fat' to 'processed and fatty meats') | No change |
| 2 | Statements about blood pressure | Statements about blood pressure | Statements about blood pressure | Minor wording change | No change |

| | | | | | |
|---|---|---|---|---|---|
| | lowering diet patterns | lowering diet patterns | lowering diet patterns | | |
| 3 | Recommended serves of vegetables | Recommended serves of vegetables | Recommended serves of vegetables | Provided a definition of a serving size | No change |
| 4 | True/False health statements | True/False health statements | True/False health statements | No change | No change |
| 5 | Cholesterol raising foods | Cholesterol raising foods | Cholesterol raising foods | Minor wording change (grilled to fried) | No change |
| 6 | Sources of healthy fats | Sources of healthy fats | Sources of healthy fats | No change | Coconut oil changed to dark chocolate as a source of saturated fat |
| 7 | Identifying foods containing fibre | Identifying grains with high fibre content | Identifying grains with high fibre content | Images of yoghurt and chicken replaced with corn flakes and rice crackers so all images from same core food group | Wording of question changed to increase complexity from "Circle the two foods with the highest fibre content" to "which of the following foods are a good source of fibre" |
| 8 | High salt foods | Protein foods high in salt | Protein foods high in salt | Images of vinegar, apple and instant noodle changed to egg, canned tuna and smoked salmon so all images from same core food group | No change |
| 9 | Alcohol content of beverages | Alcohol content of beverages | Alcohol content of beverages | Size of wine glass pictured changed to better represent text associated with the image | No change |
| 10 | Selecting meals representative of the MD | Selecting meals representative of the MD | Selecting meals representative of the MD | Text changed to highlight relevance of question to MD and cardiac health | No change |
| 11 | Core foods within the MD | Core foods within the MD | Core foods within the MD | Image of legumes further defined with text examples of legumes | No change |
| 12 | Core foods within the MD | Core foods within the MD | Core foods within the MD | No change | No change |
| 13 | Herbs/seasonings used within the MD | Herbs/seasonings used within the MD | Herbs/seasonings used within the MD | No change | No change |
| 14 | Cooking methods traditional to the MD | Cooking methods traditional to the MD | Cooking methods traditional to the MD | Wording change: "Low temperature moist cooking" changed to "moist cooking such as stewing" | No change |
| 15 | Diet patterns central to the MD | Diet patterns central to the MD | Diet patterns central to the MD | No change | No change |

| | | | | | |
|---|---|---|---|---|---|
| 16 | Diet patterns central to the MD | Selecting beverages central to the MD | Selecting beverages central to the MD | Question changed from dietary principles to question about drinks/ fluids as comments made this had not been addressed with exception of alcohol | No change |
| 17 | Selecting meals representative of the MD | Selecting meals representative of the MD | Selecting meals representative of the MD | Wording of question changed to give more context to heart health | No change |
| 18 | Selecting meals in a dine-out setting representative of the MD | Selecting meals in a dine-out setting representative of the MD | Selecting meals in a dine-out setting representative of the MD | No change | No change |
| 19 | Label reading-easy | Label reading-easy | Label reading-easy | No change | Changed from "tuna in brine and tuna in spring water" to "tuna 1 and tuna 2" to give question more complexity |
| 20 | Label Reading-Complex | Label Reading-Complex | Label Reading-Complex | Titles changed from "product" to "bread" | No change |

**Table S4.** Med-NKQ Final Version.

THE UNIVERSITY OF SYDNEY

Question 1.

Which of the following statements are true if you want to lower your blood cholesterol? You may choose more than one.

a) Eat more fruits and vegetables
b) Eat more wholegrain foods
c) Eat less fatty and processed meats
d) Eat more dairy products
e) Eat less salt on your food

Question 2.

Which of the following statements are true if you want to lower your blood pressure? You may choose more than one.

a) If using packaged foods, choose products with less salt
b) If you are overweight, try to lose some weight
c) Drink more alcohol

d) Eat more dark leafy greens
e) Eat more white bread

Question 3.

What is the recommended number of serves of vegetables per day?
Note: 1 serve= ½ cup cooked vegetables or 1 cup of raw vegetables/salad.

a) 2 serves
b) 3 serves
c) 5 serves
d) 7 serves

Question 4.

Which of the following statements are true? You may choose more than one.

a) The only nutritional benefit of fruits and vegetables is vitamins and minerals
b) Fat is always bad for your health; you should therefore avoid all fats as much as possible
c) Dietary Fibre is important for lowering your cholesterol

Question 5.

Which of the following foods are more likely to raise your blood cholesterol? You may circle more than one.

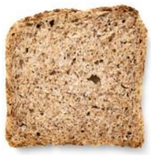

Wholemeal Bread

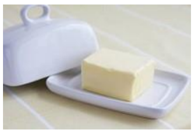

Butter

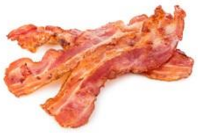

Fried Bacon

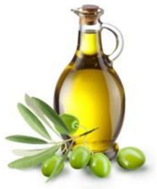

Extra Virgin Olive Oil

Question 6.

Circle the foods that are a source of healthy fats. You may circle more than one.

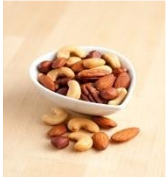

Unsalted nuts

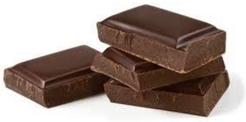

Dark Chocolate

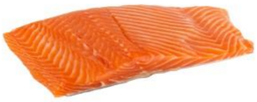

Salmon Fillet

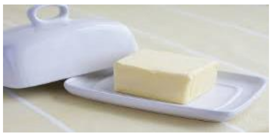

Butter

Question 7.

Which of the following foods are a good source of fibre? You may circle more than one.

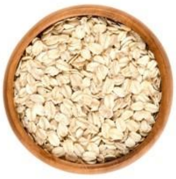

Rolled Oats

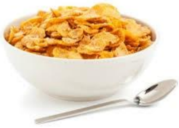

Corn Flakes

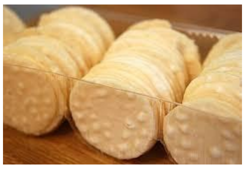

Rice Cracker

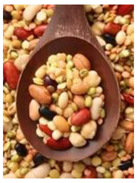

Legumes (e.g chickpeas, beans lentils)

Question 8.

Which of the following foods are high in salt? You may circle more than one answer.

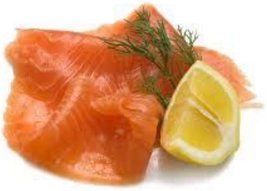

Smoked Salmon

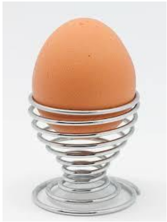

Boiled Egg

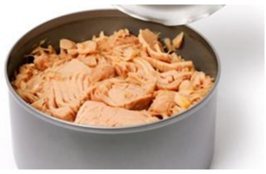

Canned Tuna in Springwater

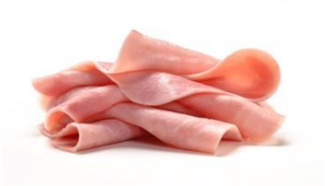

Deli Ham

Question 9.

Which alcoholic beverage below contains the lowest number of standard drinks?

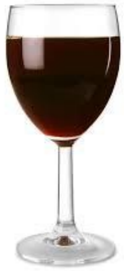

200ml Red Wine

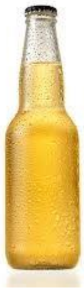

375ml full strength Beer

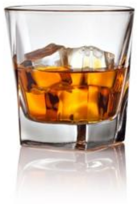

60ml of dark spirit

Question 10.

Which pasta meal better reflects the Mediterranean Diet principles for heart health? Circle the correct response.

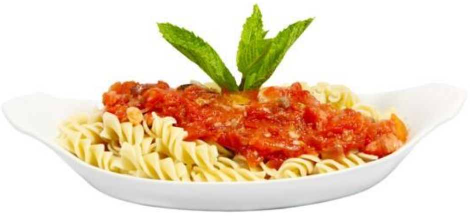

Pasta with tomato sauce

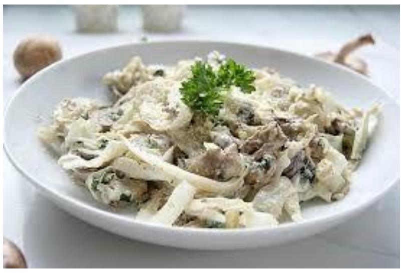

Pasta with mushroom and cream sauce

Question 11.

Which of the following foods are considered core components of the Mediterranean Diet? You may circle more than one.

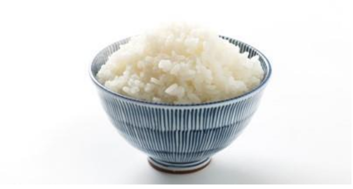

White Rice

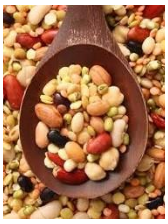

Legumes (eg chickpeas, beans, lentils)

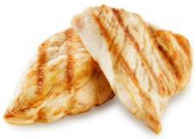

Chicken Breast

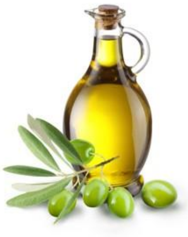

Extra Virgin Olive Oil

Question 12.

Which of the following foods are considered core components of the Mediterranean Diet? You may circle more than one.

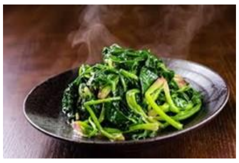

Leafy Greens

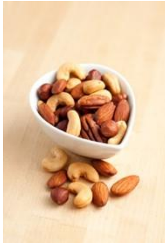

Unsalted Nuts

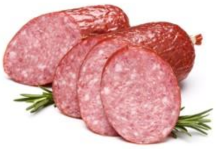

Salami

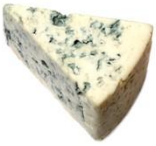

Blue Cheese

Question 13.

Which of the below are used in the Mediterranean Diet to add flavour to food? You may circle more than one.

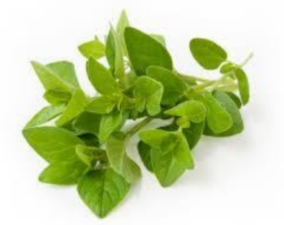

Oregano

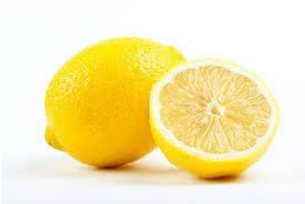

Lemon Juice

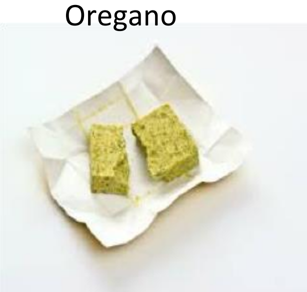

Powdered Stock Cube

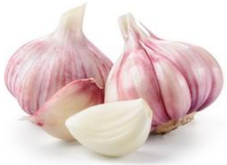

Garlic

Question 14.

Which of the following cooking methods is most typical of the Mediterranean Diet? You may choose more than one answer.

a) High Heat Stir-Fry
b) Use of moist cooking methods such as stewing
c) Barbeque

d) Only eating raw foods

Question 15.

Which of the following statements is true about the Mediterranean Diet? You may choose more than one answer.

a) Use of Extra Virgin Olive Oil as main oil.
b) Fruit Juice consumed with all meals
c) Eating red meat 3-4 times per week
d) Mostly plant based diet.

Question 16.

Which of the following drinks is consumed the most as part of the Mediterranean Diet?

a) Water
b) Fruit Juice
c) Wine
d) Coffee

Question 17.

Which of the following meals would be considered the best choice if following the Mediterranean Diet for heart health?

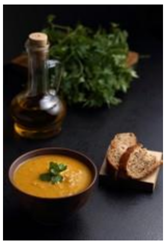

Vegetable and Lentil soup with wholegrain bread and olive oil

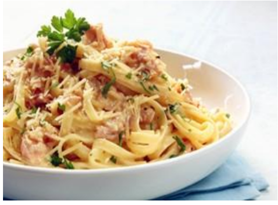

Carbonara

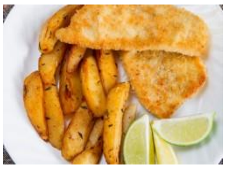

Oven baked crumbed fish and potato

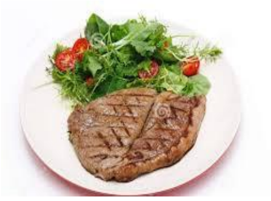

Grilled steak and garden salad with fat free dressing

Question 18.
If you were having breakfast in a cafe, which menu option would best fit the Mediterranean Diet?

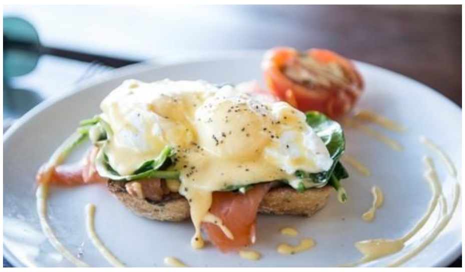

Eggs benedict with smoked salmon on toast

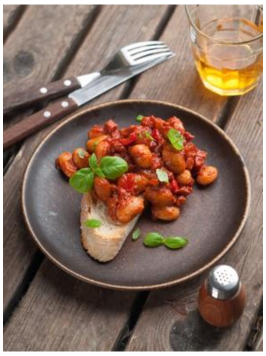

Beans cooked in tomato and onion on toast

Question 19.

Looking at products 1 & 2, which one has the most amount of salt per 100g?

## Canned Tuna 1

| Pack Size: 425g<br>Serving Size: 69g<br>Servings per packet: 4 | | |
|---|---|---|
| | Per Serve | Per 100g |
| Energy | 327kJ | 474kJ |
| Protein | 17.7g | 25.7g |
| Fat, total | 0.7g | 1.0g |
| - Saturated | 0.3g | 0.4g |
| Carbohydrate, total<br>-Sugars | <1g | <1g |
| Sodium | 311mg | 450mg |
| < means less than | | |

## Canned Tuna 2

| Pack Size: 185g<br>Serving Size: 60g<br>Servings per packet: 2 | | |
|---|---|---|
| | Per Serve | Per 100g |
| Energy | 284kJ | 474kJ |
| Protein | 15.46g | 25.7g |
| Fat, total | 0.6g | 1.0g |
| - Saturated | 0.2g | 0.4g |
| Carbohydrate, total<br>-Sugars | <1g | <1g |
| Sodium | 210mg | 350mg |
| < means less than | | |

Question 20.

Which of these two bread products is better for heart health? Circle the best choice.

## Bread 1

| Nutrition Information<br>Serving Size: 83g<br>Servings per pack: 8 | | |
|---|---|---|
| | Per Serving | Per 100g |
| Energy | 773kJ | 931kJ |
| Protein | 9.0g | 10.8g |
| Fat, total | 4.0g | 4.8g |
| -saturated | 0.5g | 0.6g |
| Carbohydrate | 25.0g | 30.1g |
| -sugars | 1.6g | 1.9g |
| Dietary Fibre | 6.1g | 7.3g |
| Sodium | 268mg | 323mg |

## Bread 2

| Nutrition Information<br>Serving Size: 60g<br>Servings per pack: 11 | | |
|---|---|---|
| | Per Serving | Per 100g |
| Energy | 612kJ | 1020kJ |
| Protein | 5.0g | 8.4g |
| Fat, total | 1.2 g | 2.0g |
| -saturated | 0.4g | 0.6g |
| Carbohydrate | 27.8g | 46.31g |
| -sugars | 1.4g | 2.4g |
| Dietary Fibre | 1.6g | 2.7g |
| Sodium | 240mg | 400mg |
